# Parent Perceptions of Changes in Child Physical Activity During COVID-19 Stay-At-Home Orders

**DOI:** 10.3389/fpubh.2021.637151

**Published:** 2021-06-07

**Authors:** Amy A. Eyler, Laurel Schmidt, Maura Kepper, Stephanie Mazzucca, Amanda Gilbert, Alan Beck

**Affiliations:** Prevention Research Center in St. Louis, Brown School, Washington University in St. Louis, St. Louis, MO, United States

**Keywords:** children, physical activity, environment, barriers, parenting, social, COVID-19

## Abstract

**Purpose:** The purpose of this study was to explore parent perceptions of changes in child physical activity during COVID-19 stay-at-home orders.

**Design:** A cross-sectional study.

**Setting:** The research team used social media, relevant organizations, and neighborhood groups to distribute the survey link in May and June of 2020.

**Subjects:** A convenience sample of parents of children aged 5–12.

**Measures:** Survey to assess parental perceptions of changes in children's physical activity before and during stay-at-home orders, and environmental and social barriers to physical activity.

**Analysis:** Results were analyzed using descriptive statistics, bivariate comparisons, and multinomial-logistic regression models with covariates of environmental factors, social factors, and frequency of factors as barriers on association with perceived physical activity change.

**Results:** Data from 245 parents were analyzed. A majority (63.7%) of parents reported a decrease in children's physical activity during stay-at-home orders. More parents indicated social barriers (e.g., lack of access to playmates) than environmental barriers (e.g., lack of access to neighborhood play spaces) to children's physical activity. In multivariate analyses, the odds of parents reporting decreased physical activity was greater for those reporting lack of playmates (OR = 4.72; 95% CI: 1.99–11.17) and lack of adult supervision (OR = 11.82; 95% CI: 2.48–56.28) as barriers. No environmental barriers were significantly associated with decreased children's physical activity.

**Conclusion:** The unique aspects of the COVID-19 pandemic provide a natural experiment for developing social and environmental strategies to improve children's overall physical activity. Assessing parental perceptions is a way to inform these future efforts.

## Introduction

Regular participation in physical activity is consistently associated with many physical and mental health benefits in children (1, 2), and there is evidence to support sustainability of these benefits into adulthood (3–5). Despite this evidence, increasing the prevalence of physical activity remains a public health challenge. The majority of children in the United States do not meet the current recommendation that children and youth should achieve at least 60 min of moderate-to-vigorous intensity physical activity each day (2, 6). Rates of physical activity among children and adolescents also vary by age and gender. There are significant decreases in physical activity with increasing age, especially for girls (7–11), with more age-related differences in elementary school than upper grade levels (10).

Restrictions due to the COVID-19 pandemic have added unique challenges to promoting physical activity in children. In an effort to prevent widespread infection of the virus, 42 states and territories issued mandatory stay-at-home orders from March 1 through May 31, 2020 (12). Places where people maintain close physical contact with one another (e.g., schools) were required to close. Since schools are a primary resource for youth activity (13), mandated closures likely impacted physical activity. Physical education and after-school sports programs were halted or limited to virtual options during this time. Opportunities for active commuting to school and recess play were also eliminated by COVID-19 stay-at-home orders. Other extra-curricular, community-based opportunities for physical activity (e.g., dance lessons, sports leagues) were halted.

Outdoor spaces such as parks and playgrounds were also closed during stay-at-home orders. Without access to these outdoor environments, children lost another important resource for physical activity (14). Lack of access to outdoor places to play may have an additional negative impact. For example there is evidence of mental health benefits of outdoor physical activity, particularly related to reduced depression and anxiety (15–17). The absence of outdoor physical activity opportunities coupled with the stressors of the pandemic may contribute to an even greater strain on mental health. Early findings report that maintaining physical activity, especially outdoors, may promote better mental and general health during periods of confinement (18–21).

Stay-at-home orders impacted families in various ways, including adding stress of employment changes as well as financial and physical health implications of the pandemic (22, 23). Many parents and caregivers had to deal with the absence of childcare and the additional task of coordinating children's online schooling. Some parents had to also juggle their own work-at-home responsibilities. This may have been especially challenging for families with younger, less independent children, resulting in modifications to usual parenting practices. For example, parents may have allowed more screen time for entertainment to accommodate inability for supervision due to work-at-home responsibilities (24).

Another way stay-at-home orders may have impacted physical activity of children is related to a change in social engagement. The pandemic created an absence of in-person social interaction with friends or extended family members. The transition from actively engaging with friends and peers (e.g., bike riding or playing outside) to socializing online likely exacerbates the negative impact of the absence of other physical activity opportunities.

The COVID-19 pandemic and subsequent stay-at-home orders created unique social and environmental situations which could impact healthy behaviors. The aims of this survey were to (1) explore parent perceptions of changes in children's physical activity during stay-at-home orders; and (2) identify social and environmental factors associated with these changes. The results from this exploratory survey can inform broader assessments to track trends over time and contribute to the development of recommendations to enhance supports for physical activity.

## Methods

### Study Design

The research team collected cross-sectional data via an anonymous online survey. This study was approved by the Washington University in St. Louis Institutional Review Board.

### Sample

Participants were recruited through convenience sampling on social media (Facebook, Twitter, and MyFitnessPal). Some platforms were geared to a broad, national audience, and others were more local. For example, survey recruitment posts were made to national research center accounts and various St. Louis neighborhood Facebook groups and advocacy groups targeted toward school age children. Sharing of the recruitment message and survey link was also encouraged. To be eligible for the study, participants had to be parents or caregivers of a child or children aged 5–12. Recruitment messages were systematically posted between May 26 and June 26, 2020, with frequencies and days of the week varying by social media platform.

### Measures

The time period of interest for the survey was March through May 2020, during state implementation of stay-at-home orders when schools and worksites were closed, and residents were asked to eliminate non-essential outings. Parents were asked to report perceptions of how their child's physical activity changed during the stay-at-home orders. The survey included questions from an existing scale of child physical activity practices (25) in addition to new questions specific to COVID-19 stay-at-home orders. See Appendix 1. The outcome variable of interest was change in child physical activity. Participants were asked “Overall, during the COVID-19 stay-at-home orders, do you feel your child's physical activity: decreased, stayed the same, or increased?” Child age and gender were also assessed. Influence of social environmental factors related to physical activity assessed in the survey were access to playmates, parent schedules, availability of adult supervision, parent interest in physical activity, support from other adults, and family support for physical activity. Influence of the physical environment assessed in the survey included access to neighborhood places for physical activity, size and layout of indoor spaces, size and layout of yard, and neighborhood safety. In order to identify perceived barriers to their child's physical activity, participants were asked to identify listed social and physical environmental factors which limited their children's physical activity. The survey was tested (*N* = 5) with parents of children in the required age range and no substantive changes were made. A link to the survey was included in recruitment messages and distributed. Parents with more than one child in the eligible age range were instructed to answer the survey based on a single child chosen at their discretion.

### Analysis

Data were analyzed with IBM SPSS 26.0 software. Significance was set at *p* < 0.05 for analyses. Of 471 survey responses, 214 were eliminated from analysis as they lacked information on either the dependent or independent variables. An additional eight cases were excluded from analysis as the children were either too young or too old for the scope of the study and four were omitted as influential outliers based on a combination of Cook's Distances in excess of 1.5, dfbetas in excess of 2, and standardized residuals in excess of 3. The final analytic sample included 245 parents.

Data were explored using descriptive statistics, frequency tables, and bivariate analyses. Correlations were assessed using Pearson's chi-square and one-way ANOVA tests.

Two multinomial logistic regressions were conducted to assess association of factors reported as barriers with change to children's physical activity. Dependent variables for the regression models were (1) decreased physical activity compared to increased or remained the same; and (2) increased physical activity compared to decreased or remained the same. The first regression included parent perception of social factors as barriers related to physical activity (availability of adult supervision, parent interest, parent schedule, support from other adults, family support for physical activity, and availability of playmates). The second included parent perception of environmental factors as barriers related to physical activity (size/layout of yard, size/layout of indoor space, neighborhood safety, and access to neighborhood play spaces). Child's age and gender were covariates controlled for in each analysis. Odds Ratios (OR) and 95% Confidence Intervals (CI) were computed. A linear regression was run to test for multicollinearity, and a Box–Tidwell test was conducted to test assumptions of linearity. Neither assumption was violated.

## Results

Descriptive statistics are presented in Table 1. The children represented in the sample were almost equal in terms of gender (51.4% male), with a mean age of 8.1 years. Most (63.7%) parents reported their child had decreased physical activity during stay-at-home orders, while 36.3% indicated physical activity stayed the same or increased.

**Table 1:** Descriptive data for demographic characteristics, physical and social environmental barriers to children's physical activity during COVID-19 stay-at-home orders.

	***N***	**%**
	245	100
**Child gender**		
Male	126	51.4
Female	119	48.6
Nonbinary/other	0	0
Missing	0	
**Child age**		
5	20	7.8
6	29	11.8
7	39	15.9
8	58	23.7
9	39	15.9
10	38	15.5
11	21	8.6
12	2	0.8
Missing	0	
**Change in child physical activity**		
Increased	49	20
Stayed same	40	16.3
Decrease	156	63.7
**Social barriers^a^**		
Availability of playmates	161	66.0
Parent schedule	134	54.9
Availability of adult supervision	70	28.7
Parent interest in physical activity	66	27.0
Availability of other adults	12	4.9
Family support for physical activity	12	4.9
Missing	0	
**Number of social barriers reported**		
0	72	29.5
1–2	140	57.2
3–4	32	13.2
5–6	1	0.04
Missing	0	
**Environmental barriers^a^**		
Access to neighborhood play spaces	102	41.8
Size/layout of indoor spaces	55	22.5
Size/layout of yard	37	15.2
Neighborhood safety	16	6.6
Missing	0	
**Number of environmental barriers reported**		
None	125	51.0
1–2	93	37.9
3–4	27	11.1
Missing	0	
**How often barriers impact physical activity**		
Rarely/never	57	23.4
Sometimes	130	53.2
Often/always	57	23.4
Missing	1	

a *Number and percent reflect a “yes” response to perception of the factor as a barrier to child physical activity*.

Availability of playmates was the most frequently reported social environment barrier to children's physical activity during stay-at-home orders (66%). Over half (54.9%) of parents reported their schedules were a barrier. Few parents (4.9%) identified support for physical activity from other adults or family members as a barrier to children's physical activity. Most parents (70%) reported at least one social environment barrier, with 13% reporting three or more. The most commonly identified environmental factor as a barrier for physical activity during stay-at-home was access to neighborhood play places (40%). The size and layout of indoor space (22%) and yard (15%) were less frequently reported environmental barriers. Neighborhood safety was indicated as a barrier to children's physical activity by 6.6% of parents. Almost half (49%) of parents reported at least one environmental factor as a barrier to their child's physical activity.

Statistically significant correlations were found between change in child's physical activity and frequency of barriers, availability of adult supervision, parent schedule, availability of playmates, size/layout of indoor space, size or lack of yard, and lack of neighborhood play space. Games–Howell *post-hoc* tests found statistically significant between-group differences between parents reporting no social barriers and those reporting two or more. There was also a statistically significant between-group difference between parents reporting no environmental barriers and those with two or more environmental barriers.

A multivariate logistic regression explored the association between change in physical activity and social barriers. Several factors emerged as significant for a decrease in child physical activity compared with no change in child physical activity (see Table 2). Perception of availability of playmates as a barrier was associated with decreased children's physical activity during COVID-19 stay-at-home orders (OR = 4.72; 95% CI: 2.00–11.17). Reporting that availability of adult supervision was a barrier for physical activity was associated with greater likelihood of reporting a decrease in children's physical activity (OR = 11.82; 95% CI: 2.48–56.28). Participants reporting their children had experienced social barriers to physical activity often or always was associated with decreased physical activity more than those reporting barriers less often (OR = 10.73; 95% CI: 1.88–61.23). In the analysis of factors associated with increased physical activity during stay-at-home orders, only one factor emerged as significant. Reporting that adult supervision was not a barrier to children's physical activity was associated with an increase in their children's physical activity (OR = 9.89; 95%CI: 1.90–51.61).

**Table 2:** Logistic regression associations of social and environmental barriers with decreased child physical activity during COVID-19 stay-at-home orders.

	**Odds ratio**	**95% Confidence intervals**	***p*-value**
**Decrease in physical activity vs. no change (*****N*** **=** **245)**			
**Demographics**			
Child age	1.305	1.02, 1.68	0.04^*^
**Child gender**			
Female	–	–	
Male	0.63	0.28, 1.41	0.26
**Social barriers^a^**			
Availability of playmates	4.72	2.00, 11.17	0.00^*^
Parent schedule	1.57	0.66, 3.70	0.30
Availability of adult supervision	11.82	2.48, 56.28	0.00^*^
Parent interest in physical activity	0.64	0.25, 1.66	0.36
Availability of other adults	0.38	0.06, 2.32	0.29
Family support for physical activity	1.05	0.07, 15.75	0.97
**Frequency of barriers to physical activity^b^**			
Rarely/never	–	–	
Sometimes	0.26	0.66, 4.76	0.26
Always/often	10.73	1.88, 61.23	0.01^*^
**Decrease in physical activity vs. no change (*****N*** **=** **245)**			
**Demographics**			
Child age	1.19	0.95, 1.49	0.13
Child gender			
Female	–	–	
Male	0.75	0.35, 1.63	0.47
**Environmental barriers^a^**			
Access to neighborhood play spaces	1.77	0.72, 4.35	0.22
Size/layout of indoor spaces	1.50	0.48, 4.70	0.49
Size/layout of yard	5.75	0.66, 50.28	0.11
Neighborhood safety	0.90	0.83, 9.74	0.93
**Frequency of barriers to physical activity^b^**			
Rarely/never	–	–	
Sometimes	2.34	0.97, 5.68	0.00^*^
Always/often	17.68	3.52, 88.80	0.00^*^

a *Participants reported a “yes” response to perception of the factor as a barrier to child physical activity*.

b *Responses to the question “During stay-at-home, how often do factors keep your child from being regularly physically active?”*.

In a multivariate logistic regression analysis of change in child physical activity and environmental factors as barriers, none were significantly associated with decrease in physical activity vs. no change in physical activity (size/layout of indoor spaces, size/presence of yard, neighborhood safety, access to neighborhood play spaces). However, higher frequency (always or often) of reported environmental factors as barriers significantly increased the odds of decreased child physical activity (see Table 3). None of the environmental factors as barriers emerged as significant when comparing increase in child physical activity and no change in physical activity.

**Table 3:** Logistic regression associations of environmental barriers with increased child physical activity during COVID-19 stay-at-home orders.

	**Odds ratio**	**95% Confidence intervals**	***p*-value**
**Increase in physical activity vs. no change (*****N*** **=** **245)**			
**Demographics**			
Child age	0.98	0.74, 1.29	0.87
**Child gender**			
Female	–	–	
Male	0.52	0.21, 1.26	0.15
**Social barriers^a^**			
Availability of playmates	1.75	0.68, 4.50	0.25
Parent schedule	0.59	0.22, 1.60	0.30
Availability of adult supervision	9.89	1.90, 51.61	0.01^*^
Parent interest in physical activity	0.44	0.14, 1.36	0.15
Availability of other adults	0.61	0.06, 5.63	0.66
Family support for physical activity	4.69	0.33, 65.89	0.25
**Frequency of barriers to physical activity^b^**			
Rarely/never	–	–	
Sometimes	0.47	0.17, 1.33	0.16
Always/often	0.68	0.08, 5.59	0.72
**Increase in physical activity vs. no change (*****N*** **=** **245)**			
**Demographics**			
Child age	0.95	0.74, 1.22	0.68
**Child gender**			
Female	–	–	
Male	0.62	0.26, 1.46	0.27
**Environmental barriers^a^**			
Access to neighborhood play spaces	1.77	0.72, 4.35	0.22
Size/layout of indoor spaces	1.50	0.48, 4.70	0.49
Size/layout of yard	5.75	0.66, 50.28	0.11
Neighborhood safety	0.90	0.83, 9.74	0.93
**Frequency of barriers to physical activity^b^**			
Rarely/never	–	–	
Sometimes	0.57	0.23, 1.44	0.24
Always/often	0.90	0.13, 6.19	0.91

a *Participants reported a “yes” response to perception of the factor as a barrier to child physical activity*.

b *Responses to the question “During stay-at-home, how often do factors keep your child from being regularly physically active?”*.

## Discussion

The quick progression of the COVID-19 pandemic resulted in many states, counties, and municipalities recommending or requiring stay-at-home orders from March through May 2020. While the stay-at-home orders are recognized as an effective strategy to prevent spread of the virus (26), it created a massive shift in work, school, and family life. The majority of parents in the current study reported a perceived decrease in physical activity of their children during this time. These results are similar to the results in the Dunton study comparing parental perception of child physical activity pre-COVID-19 pandemic (February 2020) to early COVID-19 pandemic (April through May 2020) (24). The decrease in physical activity, even if temporary, can negatively impact children's health as there is evidence to support many physical and mental health benefits of this behavior (26). Additionally, emerging research related to COVID-19 shows the protective effects of this behavior on mental health, depression, and anxiety (27–29).

This study aimed to explore physical and social environmental barriers to children's physical activity during stay-at-home orders. The most prevalent social barriers related to perceived decrease in children's physical activity were parent's schedule and lack of access to playmates. These findings concur with past research. In a systematic review of qualitative studies on barriers and facilitators to young children's physical activity, parents' busy schedule was a commonly reported barrier (30). Mailey et al. (31) also found scheduling constraints and work schedules to be barriers. These findings occurred pre-pandemic, and COVID-19 is likely placing additional strains and disruptions in work-life balance. Parents have the extra task of navigating their children's virtual learning along with their own work-at-home responsibilities (23).

Parental support is positively associated with child physical activity (26, 32). However, increased family burden due to the pandemic will likely continue as a barrier to physical activity for children. There is a need for innovative resources for parents on ways to better support their child's physical activity as the pandemic continues. Building on The Community Guide's evidence-based recommendations for family physical activity interventions (33) is a potential starting point.

Lack of access to playmates was significantly associated with a decrease in physical activity in this study, which is consistent with evidence on the positive association of engagement with others in activity and overall physical activity for children (34–36). In a systematic review on the topic, peer-to-peer participation in physical activity resulted in greater amounts of children's physical activity in six of the seven studies reviewed (37). Edwards et al. (38) reported that parents considered friends and siblings to be strong influences on their children's structured and informal physical activity. The isolating effects of stay-at-home orders impacted interactions among children. Children and adolescents virtually connected with friends during times of stay-at-home and physical distancing requirements (39). Although this mode of communication may still provide social connection, the lack of in-person interaction may also contribute to less active play together. Additionally, increased virtual communication may also increase the amount of their overall screen time and sedentary behavior, compounding subsequent negative health effects (24, 40, 41). Promoting a combination of virtual connections with ways to engage in physical activity together is a potential intervention strategy.

In addition to the social environment, the physical environment has a significant influence on physical activity. Creating and improving places for physical activity is recommended as an evidence-based strategy to increase physical activity and improve fitness (33). Having safe and accessible places to play and be active are strong correlates for child physical activity (30, 32). Stay-at-home orders eliminated opportunities in the social environment for children to be physically active, such as physical education, recess, and participation in sports programs. Additionally, recreation centers, parks, and playgrounds were closed, restricting physical environment opportunities for physical activity. We assessed the environmental factors associated with physical activity including size and layout of indoor spaces and yard, perception of neighborhood safety, and access to neighborhood places to play. The most prevalent factor reported as a barrier to child physical activity was access to neighborhood play spaces, which is not surprising given the restrictions of the stay-at-home orders. None of the individual environmental factors studied emerged as significant predictors of change in child physical activity in multi-variate analysis. However, the aggregated total of perceived barriers was a significant influence on decreased physical activity. The lack of significance of individual environmental factors is contrary to past research on the importance of home environment (30, 33, 42), yard space (43), and community resources (44) to overall child physical activity. We hypothesize our conflicting findings may be due to several elements. First, the unique impact of stay-at-home orders may have modified the perception of environment as a barrier to physical activity. Second, we did not assess specific location of participants. In doing so we may have found a skewed sample living in environments which are highly supportive for physical activity. There are substantial inequities in home, neighborhood, and community supports for physical activity across and within geographic areas (45–47). A study with a larger sample and more geographic indicators would provide broader insight into how the presence or absence of community supports impact children's physical activity.

This exploratory study provided information about child physical activity during the early phases of the pandemic. This unprecedented time period provided a unique situation for this behavior not captured previously in research. In spite of its novelty, there are several limitations which warrant mention. First, this was a cross-sectional study and causality cannot be inferred. Second, selection bias due to convenience sampling may decrease internal validity. People who would likely visit the social media sites used in recruitment may share certain characteristics compared to those who are less active on social media. Third, incomplete responses also hindered analyses. The omission of many surveys with missing data, which if completed, may have changed our results, e.g., the inability to add parent characteristics such as socio-economic status and race/ethnicity to the models due to missing data. We were unable to discern whether participants were from states without stay-at-home orders if they did not provide demographic data. Offering an incentive is a potential strategy for improving completion of surveys in future studies. Lastly, there is a chance that the drastically changed circumstances of families spending so much time together at home impacted parents' perceptions. Despite these limitations, lessons learned from methods and analyses can inform future studies.

The unique aspects of the COVID-19 pandemic provide a natural experiment for exploring the impact of the physical and social environment on children's physical activity. Assessing current perceptions of physical activity and screen time provides information for planning future studies to track sustainability of both positive and negative behavior changes over time.

## Data Availability Statement

The data generated from this study is available from the authors by request.

## Ethics Statement

The studies involving human participants were reviewed and approved by Washington University in Saint Louis Institutional Review Board.

## Author Contributions

AE: manuscript conception, design, and drafting manuscript. LS and AG: interpretation of relevant literature and data analysis. AB, SM, and MK: critical expertise and review. All authors contributed to the article and approved submitted version.

## Conflict of Interest

The authors declare that the research was conducted in the absence of any commercial or financial relationships that could be construed as a potential conflict of interest.
