# Supplementary material for: Parent Perceptions of Changes in Child Physical Activity During COVID-19 Stay-At-Home Orders
Source: Front Public Health. 2021 Jun 7;9:637151. doi: 10.3389/fpubh.2021.637151 (PMC8215440; doi:10.3389/fpubh.2021.637151)
Supplement: Supplementary file 1 [file Data_Sheet_1.DOCX]

Appendix 1

Changes in children's activity and screen time during COVID-19 stay-at-home

Questions in this survey should be answered about you and your child who is between kindergarten and 5th grade level. If you have more than one child who falls in this age range, **please select one child** to answer questions about for the remainder of the survey.

1. What is your child’s age in years?

2. What is your child’s gender? (male, female, non-binary, prefer to self-describe)

3. The questions in this section are about you, and what you think and feel. Answer each to the best of your ability. How much do you agree or disagree with each of the following statements? Mark the response which best reflects how much you agree or disagree. (Strongly disagree, disagree, somewhat disagree, neither agree nor disagree, somewhat agree, agree, strongly agree)

- Participating in regular physical activity is important to me
- Having my child be active regularly is important to me.
- I find my participation in physical activity valuable.
- I find my child’s participation in physical activity valuable.

(if somewhat agree, agree, strongly agree to any of #3 go to #4)

4. Which of the following do you find valuable about your child’s participation in physical activity during the COVID-19 stay-at-home? Please rank these from most important (1) to least important (5). You may leave items that you do not find valuable blank.

____ Energy release (e.g., “get your sillies out”)

______ Sleep quality

______ Physical health benefits (e.g., growth and development)

______ Mental health (e.g., regulating emotions)

______ Other: please describe

5. Please respond to each of the following questions about your child's physical activity and screen time behaviors to the best of your ability.

- What does your child usually do when s/he has a choice about how to spend their free time? almost always chooses sedentary activities, such as watching TV, playing video games, or reading
- usually chooses sedentary activities, such as watching TV, playing video games, or reading
- just as likely to choose physically active play as inactive recreation
- usually chooses physically active play
- almost always chooses physically active play

6. Overall, during the COVID-19 stay-at-home orders, do you feel your child’s physical activity has: (Decreased, Stayed the same, Increased)

7. On an average day, **during** the COVID-19 stay-at-home period, how many minutes does your child spend doing each of the following:

During the stay-at-home period (minutes per day)

Is this different than before stay at home? (less, same, more)

- using screens (TV, tablets, computers, smartphones, videogames) alone for entertainment
- watching TV/movies or playing video games as a family for entertainment
- using screens for educational purposes
- sitting or lying down (do not count sleep)
- playing inside
- playing outside
- participating in sports or organized activities
- playing outdoors as a form of family recreation
- using physical activity or sports as a form of family recreation, indoors or outdoors (e.g., going on a bike ride together, hiking, walking)

8. How often does your child move around, jump, or dance while watching TV or other screens? (Never, rarely, occasionally, sometimes, often, very often/always)

9. During stay-at home orders, my child’s physical activity has been limited by… (check all that apply)

- My child's lack of interest or motivation
- Lack of adult supervision
- My own lack of motivation and interest
- My busy schedule
- Other adults (for example, a co-parent) in my child's life
- Lack of support from my family, spouse, or friends
- Lack of other children to play with
- Size or layout of my indoor spaces
- Size or lack of a yard at my household
- Safety of my neighborhood
- Lack of play spaces available or open in my neighborhood

10. Please rank the answers that you selected from the previous question in order of the most (top) to least (bottom) impact on your child's physical activity right now.

______ My child's lack of interest or motivation

______ Lack of adult supervision

______ My own lack of motivation and interest

______ My busy schedule

______ Other adults (for example, a co-parent) in my child's life

______ Lack of support from my family, spouse, or friend

______ Lack of other children to play with

______ Size or layout of my indoor spaces

______ Size or lack of a yard at my household

______ Safety of my neighborhood

______ Lack of play spaces available or open in my neighborhood

11. During stay-at-home, how often do things keep your child from being physically active regularly? (Never, rarely, occasionally, sometimes, often, very often/always)

The questions in this section are about your thoughts and opinions of several topics related to your child. Answer each to the best of your ability.

- 12. How much do you agree or disagree with each of the following statements? Mark the response which best reflects how much you agree or disagree. (Strongly disagree, disagree, somewhat disagree, neither I have influence over how much physical activity my child gets
- I have influence over how much screen time (e.g., television, video games, computers, tablets) my child has agree nor disagree, somewhat agree, agree, strongly agree)

13. How much do you use your own behavior to encourage your child to be physically active?

(I don't use my own behavior to encourage my child to be active; I rarely use my own behavior to encourage my child to be active; I often use my own behavior to encourage my child to be active; I constantly use my own behavior to encourage my child to be active.)

14. Has this changed since the COVID-19 stay-at-home orders began? (No, Yes)

If yes, how has your influence changed? (I am not able to use my own behavior as much to encourage my child to be active; I am able to use my own behavior more to encourage my child to be active.)

15. On the scale provided, please indicate how often you do each of the following.

- During the COVID-19 stay-at-home, how often do you or another adult in the household… (Never, rarely, occasionally, sometimes, often)
- Has this changed since stay-at-home began? How so? (Less often now, about the same, more often now)
  - Turn on the TV, a video/movie, or a computer/tablet for your child when the weather is bad (for example, raining, too hot, too cold)?
  - Get out a toy or piece of equipment without being asked that will be used by your child during moderate or vigorous physical activity?
  - Start a physically active game with your child?
  - Send your child outside to play?
  - Turn on the TV, a video/movie, or a computer/tablet for your child so you can get things done around the house (e.g., laundry, dishes, etc.)? (5)
  - Turn on the TV, a video/movie, or a computer/tablet for your child so you can do work at home?
  - Say things to encourage your child to physical activities?
  - Say things to encourage your child to be less active (e.g., stop running)
  - Say things to encourage your child to spend less time watching TV/movies or playing video games or on a computer?

16. On the scale provided, please indicate how often you or another adult in your household talk with your child about these issues.

- How often do you or another adult in the household currently…(Never, rarely, occasionally, sometimes, often)
- Has this changed since stay-at-home began? How so? (Never, rarely, occasionally, sometimes, often)
  - discuss with your child how being physically active is good for their health?
  - discuss with your child how sedentary habits can be unhealthy?
  - discuss with your child how watching too much TV can be unhealthy?
  - discuss with your child how being physically active builds strong muscles?

17. On the scale provided please indicate how much you agree or disagree with each statement.

- During the COVID-19 stay-at-home, how much do you agree or disagree with each of the following? (Strongly disagree, disagree, somewhat disagree, agree, strongly agree, not applicable)
- Are these rules enforced differently now compared to before stay-in-place began? (Enforced less now, about the same, enforced more now)
  - My child can only play in the yard where I can see him/her from inside.
  - My child can only play in the yard when I or another adult can be outside with him/her
  - My child can only play outside our yard in the neighborhood when I can see him/her.
  - My child can only play outside our yard in the neighborhood when I or another adult is with him/her.
  - My child can only play outside our yard in the neighborhood with an older sibling is with him/her.

18. During COVID-19 stay-at-home, do you limit the amount of screen time your child has during the week (Monday through Friday)?  (No, yes)

If yes, What are the weekday limits for screen time?

If yes, Has this changed since stay-at-home orders began? (less screen time allowed now, about the same, more screen time allowed now)

19. During COVID-19 stay-at-home, do you limit the amount of screen time your child has during the weekend (Saturday and Sunday)?

If yes, What are the weekend limits for screen time?

If yes, Has this changed since stay-at-home orders began? (less screen time allowed now, about the same, more screen time allowed now)

20. How much do you agree or disagree with the following statement: It is hard to limit the amount of screen time my child has. (Strongly disagree, disagree, somewhat disagree, neither agree nor disagree, somewhat agree, agree, strongly agree)

If agree, Please explain why.

21. On the scale provided, please indicate how often you do each of the following.

- During COVID-19 stay-at-home, how often do you... Never, rarely, occasionally, sometimes, often)
- How has this changed since before stay-at-home began? (Happening less often now, happening about the same time, happening more often now)
  - offer TV, video, or movie time as a reward for good behavior?
  - take away screen time as a punishment for bad behavior?
  - use screen time to get your child to do something or to control your child’s behavior (example: You can’t watch TV until you clean up your room)?

22. When my child is bored, it helps to turn on the TV or a computer/tablet. (Strongly disagree, disagree, somewhat disagree, neither agree nor disagree, somewhat agree, agree, strongly agree)

23. What is one thing that has been positive during this time for your child’s physical activity?

24. Do you think this positive change will continue after COVID-19 stay-at-home ends? (Yes, Maybe No)

25. What school does your child attend?

26. When did your child’s school close for COVID-19 Stay at home orders?

27. Has your child’s school kept a requirement for physical education while children are not at school? (No,yes)

If yes, please describe.

28. What supports for physical activity has your child’s school distributed or promoted since closing for the COVID-19 stay-at-home? (select all that apply)

- Information about the importance of healthy physical activity and screen time behaviors
- Resources to be physically active at home
- Resources to manage appropriate screen time use
- Physical activity equipment (e.g., balls, jump ropes, rhythm scarves, etc)
- Other: please describe
- My child’s school has not offered any of these things.

29. Please answer whether the below statements are true, not true, or sometimes true about your child before and during stay-at-home orders.

- Before stay-at-home orders began, my child... (not true, sometimes true, true)
- Since stay-at-home orders began, my child... (not true, sometimes true, true)
  - felt miserable or unhappy
  - didn’t enjoy anything at all
  - felt so tired that she just sat around and did nothing
  - was very restless
  - found it hard to think properly and concentrate
  - felt lonely
  - felt irritable or angry
  - worried a lot
  - had trouble sleeping

30. Since stay-at-home orders began, my child... (not true, sometimes true, true)

- has not been content with stay-at-home orders
- was afraid of self/family/friends getting sick
- was concerned about COVID-19
- talked/asked questions about COVID-19

Please tell us about yourself and your household.

31. How old are you?

32. What is your gender? (male, female, non-binary, prefer to self-describe)

33. What is your race? (select all that apply)

- American Indian or Alaska Native; Asian, Black or African American, Native Hawaiian or Pacific Islander, White, Other please specify)

34. Would you describe yourself as Hispanic, Latino, or Spanish origin? (No, yes)

35. What is your current marital status? (Married or living with a partner, Single or never married, Divorced or separated, widowed)

36. What is your zip code?

37. Are you currently employed full time? (No, yes)

38. Are you working mostly from inside or outside your household during COVID-19 stay-at-home? (Only at home, both at home and outside of my home, only outside my home)

39. Do you share parenting responsibilities for your child with someone who lives outside of your household? (No, yes)

If yes, please describe.

40. How many people 18 years of age and older currently live in your home?

41. How many people under the age of 18 years of age , including the child you answered these questions about, currently live in your home?
